# Determinants of Caregivers' Use and Adoption of Household Water Chlorination: A Qualitative Study with Peri-urban Communities in the Peruvian Amazon

**DOI:** 10.4269/ajtmh.14-0654

**Published:** 2015-09-02

**Authors:** Jessica D. Rothstein, Elli Leontsini, Maribel Paredes Olortegui, Pablo Peñataro Yori, Pamela J. Surkan, Margaret Kosek

**Affiliations:** Department of International Health, Johns Hopkins Bloomberg School of Public Health, Baltimore, Maryland; Biomedical Research Unit, Asociación Benéfica PRISMA, Iquitos, Peru

## Abstract

The gap between the efficacy and the effectiveness of household water treatment in reducing diarrhea-related morbidity indicates the need for a better understanding of the determinants of long-term behavior change. To explore the barriers to drinking water chlorination in the Peruvian Amazon, where diarrhea is endemic among under-5 children, we conducted qualitative research with 23 caregivers from peri-urban communities of Iquitos, Peru. Our inquiry drew on the Transtheoretical Model of behavior change and the Integrated Behavioral Model for Water, Sanitation, and Hygiene to identify the most relevant contextual, psychosocial, and technological determinants of initial action and long-term adoption of chlorination. Our findings suggest that the decision to try out this practice resulted from the combined effect of knowledge of chlorination benefits and product availability and affordability. Progress from action to adoption was influenced by caretakers' understanding of dosage, the packaging of chlorine products, knowledge and skills for multipurpose laundry bleach, the taste of treated water, and reinforcement. This analysis suggests that a focus on these determinants and the household domain may help to improve the sustainability of future intervention efforts.

## Background

Diarrhea remains a major cause of under-5 morbidity in children in the developing world. Those afflicted by severe childhood diarrheal illness may endure lifelong health problems including nutritional deficits, stunted growth, decreased immune function, and impaired cognitive development.^1^,^2^ In light of the financial and political challenges of establishing centralized infrastructure for water delivery and waste removal in resource-poor settings, interim intervention activities increasingly focus on individual and household behavior change for preventing transmission of diarrhea-causing pathogens among children. The use of chlorine may interrupt several transmission pathways within the home by providing safe water for drinking and by disinfecting contaminated surfaces.

Numerous randomized controlled trials (RCTs) have demonstrated the efficacy of point-of-use water treatment (POUWT) on water quality and diarrheal disease prevention.^3^–^8^ In a systematic review of 11 RCTs, POUWT was shown to reduce the risk of diarrheal illness by 39%,^9^ and a meta-analysis of 21 studies of chlorination demonstrated a reduced childhood diarrhea risk of 29%.^10^ However, evidence for the long-term effectiveness and health impact of POUWT remains limited. Several POUWT methods have exhibited reductions in adherence over time,^11^–^14^ while studies on chlorination have shown attenuation of the reduced risk of childhood diarrhea when follow-up exceeds even short periods of only 10 weeks.^10^

The effects of surface disinfection using bleach on childhood diarrhea prevalence have not been well researched to date. Yet several studies have demonstrated the high prevalence of fecal indicator bacteria (FIB) on plates and other fomites within the home.^15^–^18^ Fecal contamination of surfaces used for food preparation (e.g., tables, plates, and cutting boards) and those frequently touched by children (e.g., floors, toys) may be a significant transmission pathway for foodborne and enteropathogens, especially because under-5 children have a great deal of hand-to-mouth exploration of areas of the household that are highly trafficked by multiple individuals.^19^

### Theoretical framework.

In their Transtheoretical Model (TTM) of behavior change, Prochaska and others (1986) postulate that when individuals engage in a new health-promoting practice, they progress through five distinct “stages of change” (pre-contemplation, contemplation, preparation, action, and maintenance).^20^,^21^ PATH (www.path.org) has adapted the TTM to capture the stages involved in adopting household water treatment and safe storage (HWTS) as: 1) *awareness* of the products, their value, and the need for HWTS; 2) *action*, including trial and current use; and 3) *maintenance*, or sustained use and purchasing behavior.^22^

The paucity of evidence for chlorination effectiveness beyond short periods suggests that interventions may have failed to address critical factors that not only motivate action but also determine the adoption and maintenance of POUWT. Less than 2% of articles identified by a recent systematic review of POUWT interventions discussed the behavioral determinants of their adoption and sustained use,^23^ pointing to a need for behavioral research on stage-specific determinants, particularly for the maintenance of water treatment behaviors.

Behavioral determinants operate in three interacting dimensions, as put forth by the Integrated Behavioral Model for Water, Sanitation and Hygiene (IBM-WASH) interventions: contextual (e.g., socioeconomic status, exposure to interventions); psychosocial (e.g., knowledge, perceived threat of disease); and technological (e.g., ease of use, strengths and weaknesses of products used for POUWT).^24^

### Study objectives.

We conducted a qualitative analysis of caregiver input regarding the barriers and facilitators to behavior change for chlorine use within the home. Our primary objectives were to assess 1) the psychosocial factors, 2) the characteristics of chlorine technologies, and 3) the contextual elements that influence caregivers' movement along the POUWT stages of change continuum. A secondary objective was to explore participants' experiences of multipurpose household bleach for surface disinfection and/or other aspects of home hygiene.

## Materials and Methods

### Setting.

The study was conducted among seven low socioeconomic status, peri-urban communities in the Peruvian Amazon's northeastern Loreto region, about 15 km southeast of the urban center of Iquitos. The participating communities consisted of one well-established township, Santa Clara, and six recently emerging settlement communities. The roads within these riverine communities are unpaved, and most houses have dirt floors and wooden walls.^24^ Roofs are generally fabricated of dried palm leaves, which must be replaced every couple of years due to annual precipitation of over 3 m; wealthier households can afford roofs of corrugated metal.^26^

Most men work in the informal sector, including agriculture, fishing, and driving *moto-carros* (a motorcycle with a passenger caravan), while the majority of women consider themselves housewives, based on the community census conducted by another study (The Etiology, Risk Factors and Interactions of Enteric Infections and Malnutrition and the Consequences for Child Health and Development [MAL-ED], see section “Sample selection”) in 2010. The six settlement communities were in various stages of development when the study was conducted: the oldest one, established in 2003, had substantial infrastructure and a dynamic economy, while a younger community, established in 2010, consisted of homes that used plastic sheets as makeshift walls and lacked electricity. According to the community census, the average household size in the study communities was approximately 5.9 individuals.

Key health indicators for this region are low, with an infant mortality rate of 43.0 (versus 16.0 per 1,000 live births nationwide) and under-5 mortality of 60.6 (versus 21.0 per 1,000 live births nationwide).^27^,^28^ Based on Peru's Demographic and Health Survey, 35.4% of children under 3 years of age in this region reported a diarrheal episode in the previous 2 weeks, as compared with 18.9% of children under 3 years nationwide.^28^ High rates of childhood stunting are closely linked to enteric infections in this population.^29^,^30^

Study communities had diverse drinking water sources: in Santa Clara, a municipal system delivered water to most households during several hour-long intervals each day, while families in the surrounding settlements shared neighborhood artesian wells or wells operated by a hand pump. It is conceivable that water sources were fecally contaminated, largely due to the widespread use of simple pit latrines in the absence of a sewage system. Water can become further contaminated upon transport to and unsafe storage in open-mouth containers within the home.^31^ A recent investigation of surface contamination in the study area revealed the presence of FIB in 86% of the tableware assessed.^18^

A unifying behavior change intervention promoting chlorination has not taken place in this region. However, information regarding this practice has been disseminated by several sources, including the health sector, radio stations, and public schools. In addition, the six settlement communities, all of which pertain to a large *Centro de Salud* (Health Center) offering comprehensive services, had access to a service run by the *Dirección de Salud Ambiental* (Environmental Health Department [EHD]) that provided free bottles of chlorine to families with children under 5 years of age in the region. This service originally required community members to bring recycled soda bottles to the clinic to be filled with chlorine, yet this system was replaced by the distribution of small chlorine bottles in 2012, when serious flooding prompted an international public health agency to donate empty bottles to the EHD. Currently, the chlorine solution is prepared and bottled at the EHD's headquarters in the urban center, and technicians from each Health Center in the region are responsible for retrieving them as needed. This service has not been available to residents of Santa Clara.

### Sample selection.

Given that women are almost exclusively responsible for home hygiene, including collecting, managing, and distributing water within the household, a sample of 23 primary caregivers were selected as the primary study participants. Participants were chosen from among the 350 caregivers whose children were enrolled in the MAL-ED study, an ongoing birth cohort study that began in 2010 to explore the synergistic relationship between malnutrition and gastrointestinal infections (mal-ed.fnih.org).

Sampling was purposive: we attempted to maximize participant diversity in terms of water management behaviors. Having conducted twice-weekly surveillance at participants' homes for 1–3 years, the cohort study field workers (FWs) were familiar with household practices, and helped to identify caregivers who took part in chlorination (users) and those who did not (nonusers) (Table 1). To ensure geographic and demographic representation, the study area was divided into four zones based on communities' shared historical and socioeconomic characteristics (with Santa Clara as Zone 1), and a minimum of four participants were recruited from each zone (Table 2). The sample size of 23 caregivers allowed us to achieve the contextual richness characteristic of qualitative studies, while also providing the opportunity to reach theoretical saturation on key topics related to our inquiry.^32^

In addition to the sample of caregivers, study participants also included the health providers in charge at both of the local health facilities as well as two EHD staff members, to provide further insight into our research questions. The health providers were identified by the field supervisor as those who were in charge at each respective facility. The EHD staff members were recruited by the researcher after visiting the EHD and explaining the study aims to several gatekeepers. Two biologists from the Basic Sanitation Unit were identified as those with the most knowledge and experience coordinating water treatment activities, and they both agreed to be interviewed.

### Data collection.

Data collection involved three qualitative research methods: in-depth interviews (IDIs), indirect observations (spot checks), and a focus group discussion (FGD) (Table 3). IDIs (*N* = 18) took place in the caregiver's home and were conducted in Spanish by a trained qualitative researcher (August–September 2013). The researcher was always accompanied by a member of the FW team, all of whom had received training in qualitative methods. Each IDI was digitally recorded and completed in approximately 30–60 minutes.

The semi-structured interview guide drew on constructs from IBM-WASH to help discover and systematize relevant barriers and facilitators for chlorination behavior change,^24^ and from the TTM adaptation^22^ to help determine each participant's stage of change. The guide also included questions surrounding additional uses of bleach within the home. Questions were informed by FW and community member input, and were pretested to ensure compatibility with the local context and vernacular.

Spot checks (*N* = 18) of the cooking area and the backyard terrain followed each IDI to assess the products used for water management (water storage containers, chlorine products). The observation checklist was derived in part from previous questionnaires used in the larger cohort study^25^ and was piloted through informal observations within community households, markets, and small shops.

One FGD (*N* = 5) was conducted at the end of the data collection with both users and nonusers from Santa Clara, and served to verify the study team's interpretations of themes that had emerged during the interviews and to elaborate on specific topics of interest.

Finally, interviews were conducted with health providers at the local health facilities (*N* = 2) and EHD staff (*N* = 2) to gather supplemental information on current and past health sector interventions for chlorination.

### Data management and analysis.

**Figure 1.**
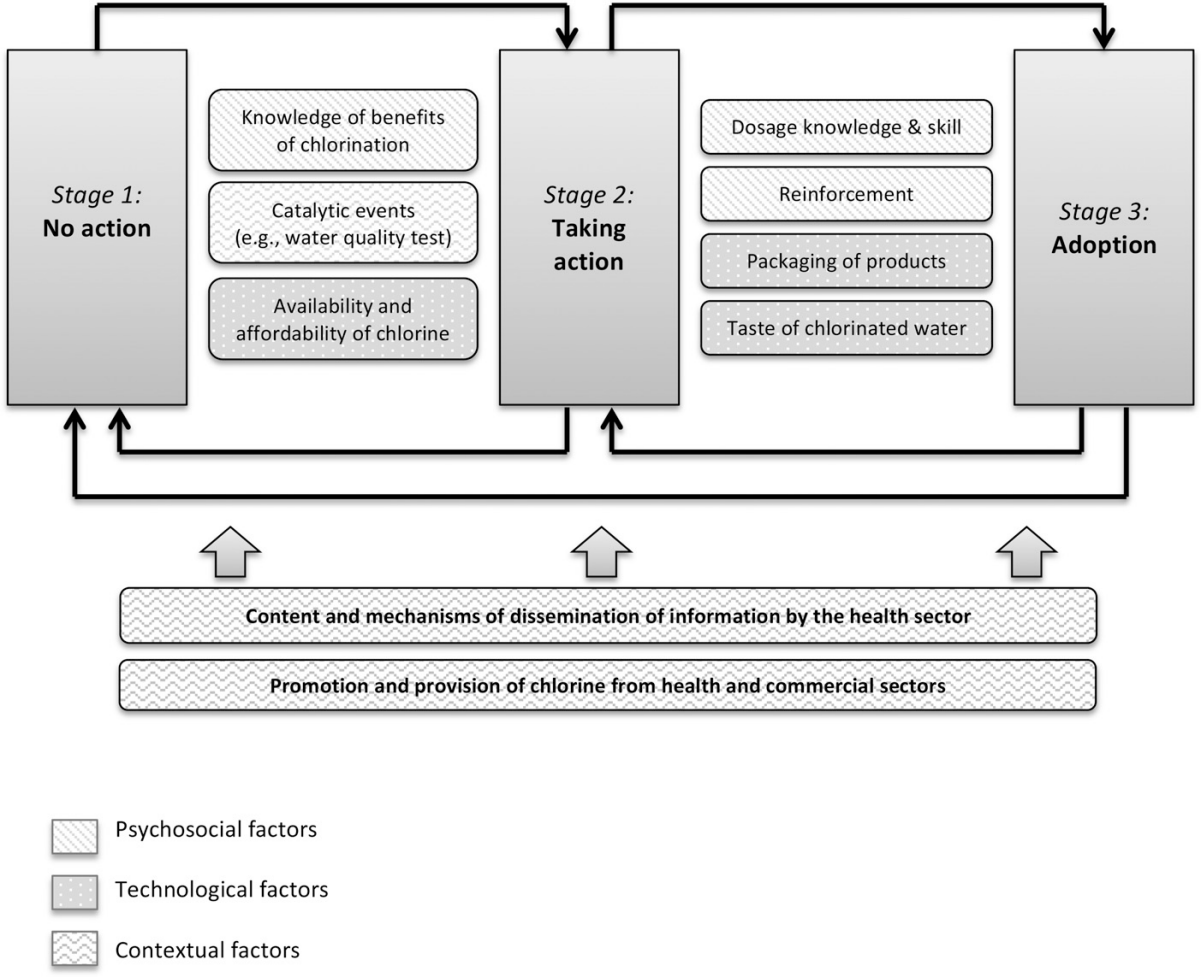
Determinants of action and adoption of point-of-use (POU) drinking water chlorination.

Digital recordings were transcribed verbatim in Spanish and supplemented with extended field notes. Quality control was ensured during transcription by reviewing random sections of the recordings with a local native speaker. Interviews were coded in Spanish by the study team using both a priori codes, drawing from IBM-WASH and the types of user groups, and emergent codes. Select sections of interviews were double-coded for quality control, and the disagreement was insignificant. Data were analyzed using an iterative process of ascertaining categories, developing a codebook, identifying key themes, and distilling the analysis to a descriptive model (Figure 1

).^33^ Ongoing discussions with the FW team throughout this process helped to validate interpretations of the data and fill in contextual gaps.

Data from household observations were entered into a data management program (Microsoft Access) and were used to triangulate with self-reported information. Baseline demographic data collected during the MAL-ED cohort study were also analyzed to provide a profile of the study participants (Table 4).

### Ethical approval.

The study protocol was approved by the institutional review boards from Johns Hopkins Bloomberg School of Public Health (Baltimore, MD) and Asociación Benéfica PRISMA (Lima, Peru). All study participants provided written informed consent.

## Results

Study participants exhibited a shared appreciation for the significance of water, as one young caregiver asserted, “Water is health. It gives life to the people.” Water was intrinsic to all of a caregiver's daily tasks: “Every morning I have to fill up *all* of my containers, and you know that water is a *necessity*, I use it to wash, to cook, to bathe, to drink—*everything*.” Nevertheless, participants' familiarity with and value for drinking water chlorination varied substantially, ranging from one caregiver who had never tasted chlorinated water to another woman who advocated water chlorination so strongly that she forbid her children from drinking water anywhere outside the home. Furthermore, participants represented several distinct positions on the POUWT behavior change continuum, with seven of the 10 nonusers having used chlorine in the past (Table 1).

### Determinants of taking action to chlorinate drinking water.

Study participants from diverse user groups described a similar set of factors that had initially motivated them to try chlorinating their household water supply.

#### Caregiver knowledge of the relationship between water, health, and chlorination.

Awareness of the risks related to water that appeared “*turbia*” (turbid) or “*sucia*” (dirty) was widespread among users and nonusers alike. The basic understanding that water treatment—both chlorination and boiling—could help to avoid diarrhea did not appear to depend on a high educational level or economic standing: it was shared by everyone from Juana,† †All names used in text are pseudonyms. a consistent user whose well-to-do family owned a large *bodega* (small convenience store), to the extremely poor Marina, a 17-year-old, illiterate mother of two children who lived in a makeshift one-room home in the youngest and most destitute settlement community (Zone 3), who occasionally chlorinated her water. Although slightly diffident during her interview, Marina stated, “if the babies drink raw water, there is diarrhea, but water with chlorine won't hurt them at all.” A smaller number of caregivers mentioned the danger of “*bichos*” (critters) and “*microbios*” (microbes) in crude water.

Participants were generally aware of the recommendations to chlorinate water used for drinking and cooking. This reflected an underlying cognizance of the importance of preventive measures, as reflected in a local saying affirmed by several interviewees: “it is better to prevent than lament.” The participants' primary source of recommendations for chlorination was the health sector: at both health facilities, participants had attended *charlas* (guided discussions) and received pamphlets from nurses or technicians in the waiting room. One caregiver who regularly treated her water explained,

Sometimes when you go to the health post, they invite you to a charla, and the nurses teach, and give examples … I always like it when they invite me to these charlas. And lots of mamás, they go … sometimes while they are at a doctor's visit, they stay to hear the recommendations … what we should do with the water, so that we stay healthy.

#### Catalytic events.

Several study participants recalled that health sector recommendations were particularly instrumental when they accompanied a specific alarming event. A child's severe episode of diarrhea was commonly cited as an incident that compelled mothers to attend the health facility, and after hearing a health provider advise water treatment in this context, several women went home and attempted chlorination for the first time. For example, Maritza recalled on taking her ill son to the Health Center two months prior, his stool sample analysis identified three different parasites. She explained,

I mostly worry for my children, and for myself, that we have to be well, we have to be healthy. That's why I started—mostly I started that day—that the biologist told me about the bichos … and if I don't do it, who else will care for my children? Who else will protect them from diseases and all that?

Another catalytic event was the performance of a water quality test by health workers (HWs) to substantiate previous claims of contaminated source water. In Zone 4, three study participants recalled initiating water chlorination after HWs had deemed the water “not fit for human consumption” in the previous year.

#### Availability and affordability of chlorine products.

The products required for chlorination were generally available and affordable for study participants. Bottles of chlorine had been offered at no cost to most households pertaining to the Health Center at one time or another, and five out of the 12 study participants who received care from the Health Center used these chlorine bottles currently or had used them in the past. Outreach HWs provided one-time distribution of chlorine directly to the homes, after which caregivers could replenish their supply by visiting the facility. However, the Health Center's stock of chlorine depended on the technician's timely transport of the products from the EHD headquarters in Iquitos, and thus the distribution system was subject to occasional bottlenecks, as mentioned by EHD and Health Center staff.

For those caregivers who did not use the free chlorine service, either by choice or by circumstance, chlorine products were widely available at a low cost throughout the study area. *Bodegas* existed along every road, and even the most limited establishments within the poorest study communities consistently offered chlorine and/or products labeled as “bleach.” The most popular local brand, Margot, was packaged into small sachets (140 g for 30 céntimos, or about 10 cents), making it accessible to some poor families that could not afford bulk products. These low prices commonly resulted in a preference for chlorination over boiling, as several participants mentioned that the routine use of charcoal or gas required for boiling water at home was more expensive than chlorine. Nevertheless, the prices of chlorine products were still perceived as prohibitive by two nonusers—a 32-year-old, unemployed, single mother of three and a 41-year-old mother of seven—who identified cost as a main barrier to chlorination.

Not all caregivers, however, were aware that commercially available bleach products for laundry and stain removal could also be used to treat water. In the sample, five current nonusers reported using Margot to wash clothes, and spot checks revealed that Margot was visible in three of these homes on the days of their IDIs.

### Determinants of adoption and maintenance.

Caregivers described a different set of processes that either encouraged or impeded their continuation of the behavior.

#### Understanding and implementing the correct dosage.

Nonusers consistently cited confusion surrounding the process of figuring out the correct dosage of chlorine for treating their water appropriately. Although some caregivers could easily recite the instructions provided at the health clinic (e.g., “ten drops of bleach” or “one teaspoon per bucket”), many others struggled to remember these details or to make sense of them once they returned home. A case in point was María, a young mother of two who had repeatedly decided to start treating her water, yet had always given it up. As she explained,

It's that I always stop—I start, but then I stop. The last time that I tried to treat my water with bleach was two months ago. My attempt was good, but I didn't know the exact dose, and added too much. And so the water tastes bad.

A related issue involved the challenges caregivers faced when they did not know the volume of their water storage containers. The buckets and pails used for drinking water varied in size and shape both between and within households. Consequently, even when caregivers understood the recommended dosage of chlorine for a given volume of water, some still struggled to determine the appropriate amount to use at home. One nonuser recalled that she had understood a nurse's directions to use “two drops per liter of water,” yet she did not know how many liters were in her household's drinking water bucket. Another occasional user explained, “Well, normally for the big bucket it's easy, but there's another bucket that is much smaller … it was hard to get the exact measurement for this one.”

#### Packaging of chlorine products.

Caregivers' level of command over chlorine dosage was also influenced by the specific design of the products. The small sachets of Margot and other local products did not come with an opening, nor did they provide a measure or dropper, and therefore did not enable accurate measurements of chlorine drops. In contrast, the free chlorine bottles provided by the Health Center were designed to allow for the bottle cap to measure the dosage.

The most common brands of chlorine and bleach products also varied in terms of the types of instructions provided on the packaging. Although both the imported Clorox bottles and the local Margot sachets offered instructions for whitening clothes, cleaning parts of the house, and disinfecting produce, only Clorox included instructions for drinking water chlorination. Margot, which was less than half the price of Clorox, was the most commonly used clothes and dish-washing agent among study participants, yet its packaging did not provide any water chlorination information. The other local brand of bleach, Shiroi, did not have any instructions at all, offering only the slogan “The queen of whitening” on its label.

In contrast, the Health Center's chlorine bottles had the advantage of offering detailed instructions for use, encompassing several types of water sources (e.g., “For water from a well or a river, use two cap-fuls of chlorine per 20 liters of water”). Several study participants lauded these improved bottles, as one caregiver asserted that treating her water was “simple” because “they've explained it—it's what it says on the bottle. *How much* we should put in. For water from the tank, it says one cap-ful.”

#### Taste of chlorinated water.

Caregivers described the unpleasant taste of chlorinated water and related grievances from their children, as a significant barrier to adopting the practice. Elva, a 40-year-old volunteer health educator who exhibited deep value for disease prevention, had attempted chlorination once, yet insisted, “It always makes your throat *burn*. I did want to do something, but we simply *cannot* be burning our *throats*!” Children's complaints over the strange flavor of the water deterred several participants' intentions to assume water chlorination. Another current nonuser who had initiated chlorination following her son's sickness explained,

But it had the taste of bleach, bleach, nothing more, and it wasn't nice for them. So, we decided to stop treating the water, and just have it that way, although … although it's not right, is it? Because if the water isn't treated, it brings certain types of illness. But … they drink it because of its natural flavor. When we add chlorine, it has another flavor, it's different ….

#### Reinforcement for habit formation.

Caregivers frequently discussed forgetting to treat their water on a daily basis and having trouble getting accustomed to the consistent use of this new practice. As one participant noted, “It's not for lack of information … but for lack of *habit*.” One explanation offered for such difficulties involved the lack of reinforcing messages on the part of the health sector. The health facilities' *charlas* were intermittent, and outreach HWs did not make regular home visits to promote chlorination for diarrhea prevention; caregivers therefore received only infrequent reminders to carry it out. For example, when asked about HW activities, 17-year-old Marina explained, “sometimes they come, and sometimes they don't.” Later in the interview, when probed on why she had not added chlorine to her water that day, she quietly uttered, “Sometimes we forget.”

A further level of complication resulted from messages that were disseminated sporadically as part of the health sector's vector control campaigns. During outbreaks of malaria and dengue, HWs would often visit households to instruct caregivers to keep their stored water protected to prevent mosquitoes from laying eggs. This advice was interpreted by some as adding chlorine, leading to message conflation of water management for mosquito reduction and diarrhea prevention. For example, when questioned about the role of outreach HWs in promoting chlorination, one nonuser pertaining to the Health Center recalled,

Now, more than a month has passed since they [the health workers] came. In the period when they were coming, it was because we [the community] were full of dengue. But then when there isn't a lot of dengue, then they don't come any more.

### Other uses of chlorine.

A small number of study participants mentioned additional ways that they used chlorine and household bleach to disinfect areas and objects within the home. Two caregivers, including one who consistently chlorinated drinking water and another who had tried in the past but discontinued, mentioned regularly adding Margot bleach to the water used while washing dishes; two others who had never practiced water chlorination also recalled doing this on occasion. Two additional consistent users had extended the use of chlorine as a disinfecting agent to other parts of the home. One young mother explained that she used Clorox, which was visible in her home on the day of the interview, to wipe down tables before eating with her toddler and teenage daughters. In addition, a middle-aged woman who often shared responsibility for her son, age 4, and grandson, age 2, recalled using the free bottle of chlorine from the Health Center to wash fruits and vegetables, and to disinfect the children's toys on a daily basis. As she explained,

Everyday I get all of the toys together, putting them in a little bucket with …chlorine, and I wash them nice and I dry them….Everything that is on the floor, he puts everything that he grabs in his mouth, it all goes in his mouth. And I say, this is why he has diarrhea, because everything that he grabs, he puts in his mouth!

Nevertheless, awareness of the benefits of using chlorine to disinfect surfaces and objects in the home was far less common than the general knowledge for the recommended practice of drinking water chlorination. For those women who did engage in these practices, they did not recall receiving any type of promotional messages for them from the health sector; rather, they had adopted these behaviors of their own accord.

## Discussion

Our qualitative research with caregivers in peri-urban Peru demonstrates the distinctions between the motivators of action for chlorination and those factors influencing the potential for its adoption. Although the combined effect of caregiver knowledge, product availability, and catalytic events may prompt initial attempts at chlorination, several barriers threaten the practice's sustainability.

Figure 1 provides an explanatory model, based on the data, which identifies the most salient determinants of movement along the POUWT behavior change continuum. These factors may motivate progress (e.g., if the caregiver's “dosage knowledge and skill” is *high*, it will facilitate the move from action to adoption) or impede it (e.g., if the “dosage knowledge and skill” is *low*, the caregiver will likely remain an inconsistent user or relapse to nonuse). The bidirectional arrows represent the likelihood of recidivism to an earlier stage. Figure 1 demonstrates how caregiver capacity to adopt chlorination was limited by several attributes specific to the chlorine products, as well as psychosocial factors related to knowledge, skill, and habituation. Figure 1 also captures the underlying contextual factors influencing progress along the behavior change continuum, which consist of activities implemented by the health and commercial sectors to disseminate information as well as chlorine products.

Several determinants mediating progress between action and adoption depend on how resources available within the contextual dimension translate to the household, a process first characterized by Berman and others (1994) as the “household production of health” (HHPH). This involves “a dynamic behavioral process” in which external technologies and information interact with households' internal knowledge, resources, and customary practices in the context of health promotion.^34^

With regard to the circulation of information, we have seen that raising awareness about the risks of crude water and the benefits of chlorination inspire its initial use, particularly when the health sector relays such recommendations in response to a catalytic event, such as during a child's illness or while disseminating the results of water quality testing. Yet the fact that this information is disseminated primarily in the public arena results in gaps in another area of knowledge—precisely *how* to carry out the chlorination process within the home environment. The incomplete understanding and skills for measuring chlorine dosage and water volume often precluded the adoption of the practice. Furthermore, the need for positive reinforcements and the lack of reminders at home limited caregivers' capacity to assume a new daily habit.

Similarly, the ubiquitous presence of commercially available or no-cost technologies throughout the study communities and the facility with which caregivers could obtain them, did not ensure their ease of use within the home. Specific obstacles included the absence of design features to allow for accurate measurements and of instructions to guide the behavior. These shortcomings also translated to a lack of awareness of the multipurposing capabilities of bleach products among some caregivers, limiting their capacity to initiate drinking water chlorination or use bleach marketed for laundry in the absence of the free chorine bottles from the health center. In these ways, our data has demonstrated that while the decision to initiate chlorination is closely linked to events taking place in the public arena, the progression to adoption is shaped largely by processes that unfold within the home environment.

There are several mechanisms by which the confluence of these multidimensional determinants in Figure 1 may preclude chlorination adoption and maintenance. First, caregivers' confusion surrounding the appropriate dosage for the household's water supply likely resulted in low self-efficacy (i.e., a person's conviction that they can successfully perform a specific behavior and achieve certain goals) for successfully chlorinating one's water. Bandura and others (1997) describe self-efficacy as the most important predictor of preventive behaviors.^35^ For our study participants, children's complaints about the taste of chlorine and the lack of alternative support structures may have further weakened caregivers' self-efficacy for the new practice, diminishing their motivation to overcome other barriers to chlorination.

Second, the determinants that emerged from our data may have created obstacles for habituation, which plays a critical role in the adoption of WASH-related behaviors that must be performed on a daily basis to affect health outcomes. Wood and others^37^ posit that habit formation is inextricably linked to a “stable context,” which enables the regular repetition of the behavior and offers essential “contextual cues.” In our study, the absence of appropriate health sector–related reminders at the household level appears to have created an unstable context that did not support habituation of chlorination. Our findings reflect the conclusions of previous studies on hand washing, which identified the irregular availability of soap and the absence of parents' reminders at home as barriers to maintenance.^38^,^39^

Our findings have several implications for future interventions. First, the technologies must be designed so as to single-handedly equip users to appropriately carry out the practice. Just as the free chlorine bottles from the Health Center provide measuring caps, promotion of commercially available bleach products should include a dropper or measure as well as instructions for water chlorination to reduce uncertainties surrounding the correct dosage. Second, chlorine provision or promotion should be coupled with the provision or promotion of standardized durable containers with tight-fitting lids so as to eliminate confusion regarding water volume and dosage. The potential of this intervention strategy is supported by previous research conducted with this population that demonstrated a 31.8% decreased incidence of *Shigella* among children living in homes where water storage containers had tight-fitting lids.^25^ Provided that containers are available for 3–4 USD in this area, their provision or promotion as an intervention component would not be a large additional expense in our study communities or similar locales. Even if the bucket were to be lost or damaged over time, the distribution of a container labeled with the volume would be expected to orient and improve caregivers' future volumetric assessments.

Third, the chlorine packaging of the intervention should specify the concentration of chloride within the bottle, along with common bleach products with the same concentration. This would allow for caregivers to easily refill the Health Center's bottle with inexpensive products that may be locally purchased or to repurpose laundry bleach with high self-efficacy, so that processes of habit formation are not interrupted if there is a bottleneck in the distribution system or other barriers to replenishing one's supply. However, it would be important for the intervention to be based on careful assessment of popular local products, since chloride concentrations may differ (e.g., Clorox has 4.9% sodium hypochlorite, while Margot has 7.5%, and quality control of true concentrations differs depending on the reported product uses, with standards for water treatment being the most regulated).

Finally, behavior change communication activities for chlorine use should concentrate on capacity building at the household level, and thereby treat the household as the “institutional focus,” as suggested by HHPH.^34^ By making regular home visits, outreach HWs could measure the volume of existing containers and provide personalized guidance on the respective chlorine dosage. This would also provide opportunities for HWs to model the chlorination process and provide encouragement to increase caregiver self-efficacy and to provide regular reminders to ensure a stable context for habit formation. Several study participants voiced a desire for regular home visits, suggesting that the acceptability of this intervention component would be high. As one occasional user insisted, “They [the health workers] should come around once a week so that people, parents, always keep it in mind that they have to treat their water.”

This may involve health sector efforts to establish uniform messages regarding uses of chlorine for dissemination by different programs. For example, in the Peruvian Amazon, the health sector could leverage other government-sponsored outreach programs (e.g., Primera Infancia) to communicate reminders to households with young children. In addition, vector control campaigns must more explicitly clarify the difference between chlorinating drinking water and protecting stored water from mosquitoes, to avoid the situation in which mixed messages lead some caregivers to treat their water on a seasonal or situational basis, rather than adopting it as a regular practice.

The health sector may also increase its role in motivating caregivers to initiate and sustain POUWT by modifying their perceptions of existing water quality through continued water quality testing. The association between the perceived lack of safety of drinking water supply and a household's demand for technologies to improve water quality has been demonstrated in other settings.^40^ This suggests that educational activities to address misconceptions about water safety may play an important role in behavior change communication. Provided that several study participants recalled the significance of water quality tests, the expansion of these tests and the targeted delivery of their results may serve as a powerful avenue for a health sector informational campaign.

Another promising addition to outreach activities would involve the concurrent promotion of chlorine use for surface disinfection. The International Scientific Forum on Home Hygiene champions surface disinfection as an important practice for reducing the transmission of diarrhea-causing pathogens within the home.^41^ Provided that some study participants were already doing this on their own initiative, it may be appropriate for HWs to emphasize that the same technology can provide added benefit for children's health if used to disinfect dishes, tables, toys, and other surfaces in the home. In conjunction with these messages, HWs should also ensure that caregivers are aware that the bleach products that they may already have at home for washing and whitening their clothes can also serve as technologies for water chlorination and surface disinfection.

A difficult challenge to address is the taste of chlorine, as this is an inherent feature of the technology that requires acclimatization. Chlorine flavor sensitivity has been cited as a major barrier to the consumption of chlorinated water in contexts throughout the world.^42^–^45^ However, there is some evidence suggesting that the acceptability of the chlorine taste in drinking water improves as exposure to it increases.^46^ This suggests that household members' aversion to chlorinated water will subside over time and that interventions should focus on ensuring that the motivations for POUWT outweigh the unpleasant taste of chlorine during the early stages of behavior change.

At the same time, it is possible that efforts to ensure appropriate dosing through modifications to technologies and/or HWs' messages may improve the taste of treated water, since overdosage exacerbates the intensity of chlorine flavor.^46^ Our study also provides some evidence that HWs may help to develop strategies for appeasing children's reactions to the unpleasant smell and taste of chlorinated water. One participant recalled that a HW had advised her to chlorinate the water while her children were playing outside; this effort to divert attention away from the practice ultimately lessened her children's complaints.

Finally, there are several other barriers to the adoption of water chlorination that were not expressed by our study participants, but which may play an important role in other resource-poor settings. In areas where alternative POUWT practices, such as filtration or boiling, have been heavily promoted in the past, several additional variables may influence consumer preferences for drinking water chlorination. For example, in their study of households' evaluations of water filters as compared with chlorine packets in India, Poulos and others^47^ identified the amount of time required to treat water and the convenience of the retail outlets where products were available as important determinants of use. In this way, the perceived advantages or disadvantages to chlorination will vary based on specific contextual factors. In addition, the price of chlorine technologies may serve as a significant barrier in areas where the products are not provided free of charge by the health sector or widely available at a low cost, as they were in our study site. Finally, although perceived social norms and social desirability did not emerge as important themes during our data collection, these factors may significantly impact caregivers' motivation to take up POUWT in other settings.^48^,^49^

### Limitations.

The study could have been limited by the following. First, self-reported behaviors may be subject to social desirability bias, thus IDI participants may have overreported the frequency and consistency of chlorination.^50^ However, this bias was likely minimized by the fact that neither this study nor the larger cohort study was associated with the promotion of a technology. In addition, the interviewer was trained to interact in a nonjudgmental manner, and the caregivers were accustomed to providing candid and genuine responses during the interviews, after several years of participation in the larger cohort study.^25^,^29^,^30^

Second, the fact that our sample was based in a specific setting in the Peruvian Amazon may limit the transferability of the findings. However, our study area may be comparable to peri-urban communities in other parts of Peru or Latin America. This is significant in view of the fact that peri-urban areas are expanding rapidly in the developing world, and their lack of infrastructure for water and sanitation call for improved behavioral interventions.^51^

## Conclusions

Our results suggest that the sustainability of interventions promoting chlorination requires a “stage-matched” approach informed by a nuanced understanding of the determinants of chlorine use and adoption.^21^ The use of a behavioral ecological framework such as IBM-WASH, which explicitly includes technological factors, may facilitate such an understanding.^24^ In addition, a focus on the household environment and caregiver capacity through home visits may be a key component of effective intervention design. These steps hold great promise for moving us closer to realizing the full health impact of chlorine use within the home.

## Figures and Tables

**Table 1:** POUWT practices of study participants

Category*	IDI participants (*N* = 18)	FGD participants (*N* = 5)	Total (*N* = 23)
Current users			
Consistent users of chlorine	5	2	7
Occasional users of chlorine	3	0	3
Current nonusers			
Tried in past and discontinued; does not currently boil water	4	1	5
Tried in past and discontinued; currently boils water	2	1	3
Has never used chlorine; currently boils water	1	0	1
Has never used chlorine nor boiled water in past	3	1	4

FGD = focus group discussion; IDI = in-depth interview; POUWT = point-of-use water treatment.

* Category defined by user self-report.

**Table 2:** Data collection zones

Zone	Data collection	Water source	Health facility	Relevant characteristics
1	3 pilot IDIs	Piped to home	Limited facility in town (Health Post)	Established 1949; centralized water treated with flocculation and chlorination available to most homes
	3 IDIs			
	1 FGD			
2	4 IDIs	Artesian well; well with hand pump	Larger facility between 1 and 5 km from communities (Health Center)	Established 2002; first unauthorized settlements in area; population close to 1,000 individuals
3	4 IDIs	Artesian well; well with hand pump		Established 2010–2011; smaller villages with less infrastructure; more severe poverty
4	4 IDIs	Well with hand pump		Established 2003–2010; some participants (*N* = 2) lacking electricity

FGD = focus group discussion; IDIs = in-depth interviews.

**Table 3:** Data collection methods and instruments

Data collection method	Goal of method	Development of instrument
IDI with caregivers	Explore POUWT behaviors, how they have changed over time, and what factors motivate or impede the changes	Informed by behavior change continuum and IBM-WASH; modified based on input from FWs and community members
Indirect observation	Assess products in home as proxy for behaviors and evaluate access to resources	Informed by observations of community members and *bodegas* before formal data collection, and surveys from larger cohort study
Focus group discussion	Validate interpretation of themes arising from analysis of IDIs	Created during iterative process of data collection and analysis
Interview with health sector staff	Gather supplemental information on current and past health sector interventions for chlorination	Questions emerged in response to themes mentioned during IDIs

FWs = field workers; IBM-WASH = Integrated Behavioral Model for Water, Sanitation, and Hygiene; IDI = in-depth interview; POUWT = point-of-use water treatment.

**Table 4:** Demographic information of IDI participants

Category	*N*
Age (years)	
≤ 19	1
20–24	3
25–29	7
30–34	4
≥ 35	3
Marital status	
Never married	2
Married	15
Divorced	1
Education level	
Incomplete primary (< 6 years)	4
Complete primary (6 years)	2
Incomplete secondary (< 14 years)	11
Complete secondary (14 years)	1
Number of children	
1–2	9
3–5	7
≥ 6	2
Main source of drinking water	
Borehole	7
Artesian well	5
Piped to home	5
Other	1
Daily income per capita (USD/day)	
< 1	10
1–2	7
> 2	1

IDI = in-depth interview; USD = U.S. dollars.
